# Lutetium(III) cyclo­tetra­phosphate

**DOI:** 10.1107/S1600536810016363

**Published:** 2010-05-08

**Authors:** Aïcha Mbarek, Mohieddine Fourati, Daniel Zambon, Daniel Avignant

**Affiliations:** a Laboratoire de Chimie Industrielle, Département de Génie des Matériaux, Ecole Nationale d’Ingénieurs de Sfax, Université de Sfax, BP W 3038, Sfax, Tunisia; b Laboratoire des Matériaux Inorganiques, UMR CNRS 6002, Université Blaise Pascal, 24 Avenue des Landais, 63177 Aubière, France

## Abstract

Single crystals of the title compound, tetra­lutetium(III) tris­(cyclo­tetra­phosphate), Lu_4_(P_4_O_12_)_3_, were obtained by solid-state reaction. The cubic structure is isotypic with its Al^III^ and Sc^III^ analogues and is built up from four-membered (P_4_O_12_)^4−^ phosphate ring anions (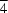 symmetry), isolated from each other and further linked through isolated LuO_6_ octa­hedra (.3. symmetry) *via* corner sharing. Each LuO_6_ octa­hedron is linked to six (P_4_O_12_)^4−^ rings, while each (P_4_O_12_)^4−^ ring is linked to eight LuO_6_ octa­hedra.

## Related literature

The title compound belongs to a structural type discovered a long time ago through the Al_4_(P_4_O_12_)_3_ member, the structure of which was first investigated by Hendricks & Wyckoff (1927 ▶) and then described by Pauling & Sherman (1937 ▶). Since then, five isotypic compounds have been characterized: Cr_4_(P_4_O_12_)_3_ (Rémy & Boullé, 1964 ▶); Ti_4_(P_4_O_12_)_3_ (Liebau & Williams, 1964 ▶); Fe_4_(P_4_O_12_)_3_ (d’Yvoire *et al.*, 1962 ▶); Sc_4_(P_4_O_12_)_3_ (Bagieu-Beucher, 1976 ▶; Mezentseva *et al.*, 1977 ▶; Bagieu-Beucher & Guitel, 1978 ▶; Smolin *et al.* 1978 ▶) and Yb_4_(P_4_O_12_)_3_ (Chudinova, 1979 ▶). For a review of the crystal chemistry of cyclo­tetra­phosphates, see: Durif (1995 ▶). For other polymorphs of composition Lu(PO_3_)_3_, see: Höppe & Sedlmaier (2007 ▶); Yuan *et al.* (2008 ▶); Bejaoui *et al.* (2008 ▶).

## Experimental

### 

#### Crystal data

Lu_4_(P_4_O_12_)_3_
*M* *_r_* = 1647.52
Cubic, 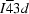

*a* = 14.6920 (6) Å
*V* = 3171.3 (2) Å^3^
*Z* = 4
Mo *K*α radiation
μ = 13.08 mm^−1^
*T* = 296 K
0.18 × 0.10 × 0.08 mm

#### Data collection

Bruker APEXII CCD diffractometer
Absorption correction: multi-scan (*SADABS*; Bruker, 2008 ▶) *T*_min_ = 0.534, *T*_max_ = 0.746
3088 measured reflections
717 independent reflections
659 reflections with *I* > 2σ(*I*)
*R*_int_ = 0.034

#### Refinement

*R*[*F* ^2^ > 2σ(*F* ^2^)] = 0.020
*wR*(*F* ^2^) = 0.038
*S* = 1.03
717 reflections
41 parameters
Δρ_max_ = 0.90 e Å^−3^
Δρ_min_ = −0.67 e Å^−3^
Absolute structure: Flack (1983 ▶), 272 Friedel pairs
Flack parameter: 0.000 (15)

### 

Data collection: *APEX2* (Bruker, 2008 ▶); cell refinement: *SAINT* (Bruker, 2008 ▶); data reduction: *SAINT*; program(s) used to solve structure: *SHELXS97* (Sheldrick, 2008 ▶); program(s) used to refine structure: *SHELXL97* (Sheldrick, 2008 ▶); molecular graphics: *CaRine* (Boudias & Monceau, 1998 ▶) and *ORTEP-3* (Farrugia, 1997 ▶); software used to prepare material for publication: *SHELXTL* (Sheldrick, 2008 ▶).

## Supplementary Material

Crystal structure: contains datablocks I, global. DOI: 10.1107/S1600536810016363/wm2342sup1.cif

Structure factors: contains datablocks I. DOI: 10.1107/S1600536810016363/wm2342Isup2.hkl

Additional supplementary materials:  crystallographic information; 3D view; checkCIF report

## Figures and Tables

**Table 1:** Selected bond lengths (Å)

Lu—O3^i^	2.182 (3)
Lu—O3^ii^	2.182 (3)
Lu—O3	2.182 (3)
Lu—O2^i^	2.185 (4)
Lu—O2^ii^	2.185 (4)
Lu—O2	2.185 (4)
P—O2^iii^	1.464 (4)
P—O3	1.481 (4)
P—O1^iv^	1.583 (3)
P—O1	1.594 (3)

Symmetry codes: (i) 
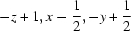
; (ii) 
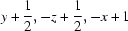
; (iii) 
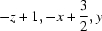
; (iv) 
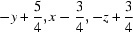
.

**Fig. 1.**
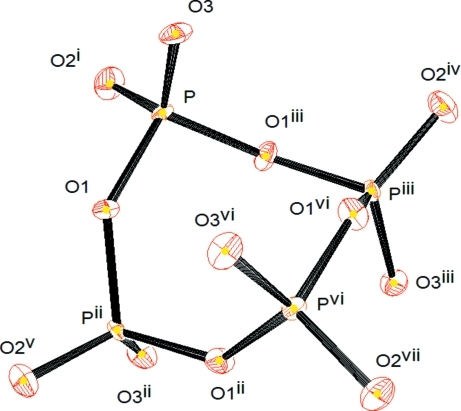
ORTEP-3 view of the four-membered phosphate (P4O12)4- ring anion. Displacement ellipsoids are drawn at the 50% probability level. Symmetry codes: (i) 1-z, 3/2-x, y ; (ii) 3/4+y, 5/4-x, 3/4-z ; (iii) 5/4-y, -3/4+x,3/4-z ; (iv) -1/4+x, 1/4-z, 3/4-y ; (v) 9/4-x, 1/4+z, 3/4-y ;(vi) 2-x, 1/2-y, z ; (vii) 1+z, -1+x, y.

**Fig. 2.**
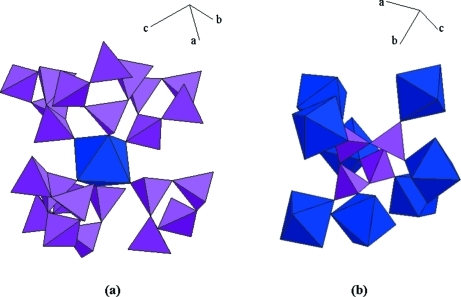
Partial view of the Lu4(P4O12)3 structure showing: (a) the connections between the LuO6 octahedron and the (P4O12)4- ring anions, (b) the connections between the (P4O12)4- ring anion and the LuO6 octahedra.

## supplementary crystallographic information

### Comment

The title compound is the third polymorph of composition Lu(PO_3_)_3_ besides the monoclinic form described by Höppe & Sedlmaier (2007) and Yuan *et al.* (2008) and the trigonal form more recently reported by Bejaoui *et al.* (2008). The title compound is also the less dense polymorph with a calculated density of 3.451 Mg^.^m^3^ versus 3.587 Mg^.^m^3^ for the trigonal and 3.708 Mg^.^m^3^ for the monoclinic form and is probably the highest temperature form. This cyclotetraphosphate belongs to a structural type (cubic, with space group *I*43*d*) known since 1927 through the archetype Al_4_(P_4_O_12_)_3_ determined by Hendricks & Wyckoff (1927). Then Pauling & Sherman (1937) gave the first description of the structure and reported roughly estimated atomic coordinates deduced from geometrical considerations. Since this time only five members of this family, viz. Cr_4_^III^(P_4_O_12_)_3_ (Rémy & Boullé, 1964), Ti_4_^III^(P_4_O_12_)_3_ (Liebau & Williams, 1964), Fe_4_^III^(P_4_O_12_)_3_ (d'Yvoire *et al.*,  1962), Sc_4_^III^(P_4_O_12_)_3_ (Bagieu-Beucher, 1976; Mezentseva *et al.*,1977 and Smolin *et al.*, 1978) and Yb_4_^III^(P_4_O_12_)_3_ (Chudinova, 1979), have been identified. Corresponding unit cell parameters are listed in Durif (1995). Among these isotypic compounds only the structure of the Sc_4_(P_4_O_12_)_3_ cyclotetraphosphate has almost simultaneously been refined from single-crystal data by Bagieu-Beucher & Guittel (1978) and Smolin *et al.* (1978). Their refinements confirmed the description of Pauling & Sherman (1937) according to which all the crystallographically independent atoms except the *A*^III^ element (.3. symmetry) are in general positions. The structure is built of four-membered phosphate ring anions (P_4_O_12_)^4-^ (Fig. 1), isolated from each other and further linked by LuO_6_ octahedra by sharing corners. Each LuO_6_ octahedron is linked to six (P_4_O_12_)^4-^ rings (Fig. 2a) while each (P_4_O_12_)^4-^ ring is linked to eight LuO_6_ octahedra (Fig. 2b) through oxygen atoms with shorter P—O distances (1.464 (4) and 1.481 (4) Å). The (P_4_O_12_)^4-^ ring anions are located around the 12a Wyckoff positions of space group *I*43*d* and exhibit 4 symmetry. Comparison of the (P_4_O_12_)^4-^ ring anions in both Sc_4_(P_4_O_12_)_3_ and Lu_4_(P_4_O_12_)_3_ structures shows these two ring anions being geometrically quite identical with alternating upward- and downward-pointing tetrahedra and P—O—P angles of 137.1° and 136.9 (2)°, respectively. The P—O distances in the PO_4_ groups are identical within their e.s.d.. The four bridging oxygen atoms of these ring anions are located at the apices of a flattened tetrahedron with characteristic angles of 148.22° and 94.30° for Sc and 147.95° and 94.37° for the Lu cyclotetraphosphate. The LuO_6_ octahedron is very slightly distorted along a threefold axis, resulting in two sets of Lu—O distances equal to 2.182 (3) and 2.185 (4) Å, respectively.

### Experimental

Single crystals of the title compound were obtained by solid state reaction while attempting to synthesized a long chain polyphosphate by reacting Lu_2_O_3_ with (NH_4_)H_2_PO_4_ and Rb_2_CO_3_ in an alumina boat. A mixture of these reagents in the molar ratio 27 : 85.5 : 8.7 was used for the synthesis. The mixture was successively heated at 473 K for 24 hours, then at 573 K for 24 additional hours and finally at 813 K for 24 hours. Then the sample was cooled down to 683 K at the rate of 3 K h^-1^ and maintained at this temperature for 36 hours. Finally, the sample was cooled down to room temperature by shutting the muffle furnace off. Single crystals were extracted from the batch by washing with hot water and filtering. The crystals were dried at 353 K in an oven. A translucent octahedral crystal of the title compound was selected for the structure refinement.

### Refinement

The highest residual peak in the final difference Fourier map was located 0.87 Å from atom Lu and the deepest hole was located 0.99 Å from atom Lu.

### Crystal data

**Table:**

Lu_4_(P_4_O_12_)_3_	*D*_x_ = 3.451 Mg m^−^^3^
*M**_r_* = 1647.52	Mo *K*α radiation, λ = 0.71073 Å
Cubic, *I*43*d*	Cell parameters from 1548 reflections
Hall symbol: I -4bd 2c 3	θ = 3.4–30.3°
*a* = 14.6920 (6) Å	µ = 13.08 mm^−^^1^
*V* = 3171.3 (2) Å^3^	*T* = 296 K
*Z* = 4	Truncated octahedron, colourless
*F*(000) = 3008	0.18 × 0.10 × 0.08 mm

### Data collection

**Table:**

Bruker APEXII CCD diffractometer	717 independent reflections
Radiation source: fine-focus sealed tube	659 reflections with *I* > 2σ(*I*)
graphite	*R*_int_ = 0.034
Detector resolution: 8.3333 pixels mm^-1^	θ_max_ = 30.4°, θ_min_ = 3.9°
φ and ω scans	*h* = −16→11
Absorption correction: multi-scan (*SADABS*; Bruker, 2008)	*k* = −6→20
*T*_min_ = 0.534, *T*_max_ = 0.746	*l* = −19→9
3088 measured reflections	

### Refinement

**Table:**

Refinement on *F*^2^	Primary atom site location: structure-invariant direct methods
Least-squares matrix: full	Secondary atom site location: difference Fourier map
*R*[*F*^2^ > 2σ(*F*^2^)] = 0.020	*w* = 1/[σ^2^(*F*_o_^2^) + (0.0088*P*)^2^] where *P* = (*F*_o_^2^ + 2*F*_c_^2^)/3
*wR*(*F*^2^) = 0.038	(Δ/σ)_max_ < 0.001
*S* = 1.03	Δρ_max_ = 0.90 e Å^−^^3^
717 reflections	Δρ_min_ = −0.67 e Å^−^^3^
41 parameters	Absolute structure: Flack (1983), 272 Friedel pairs
0 restraints	Flack parameter: 0.000 (15)
0 constraints	

### Special details

**Table:**

Geometry. All esds (except the esd in the dihedral angle between two l.s. planes) are estimated using the full covariance matrix. The cell esds are taken into account individually in the estimation of esds in distances, angles and torsion angles; correlations between esds in cell parameters are only used when they are defined by crystal symmetry. An approximate (isotropic) treatment of cell esds is used for estimating esds involving l.s. planes.
Refinement. Refinement of *F*^2^ against ALL reflections. The weighted *R*-factor wR and goodness of fit *S* are based on *F*^2^, conventional *R*-factors *R* are based on *F*, with *F* set to zero for negative *F*^2^. The threshold expression of *F*^2^ > σ(*F*^2^) is used only for calculating *R*-factors(gt) etc. and is not relevant to the choice of reflections for refinement. *R*-factors based on *F*^2^ are statistically about twice as large as those based on *F*, and *R*- factors based on ALL data will be even larger.

### Fractional atomic coordinates and isotropic or equivalent isotropic displacement parameters (Å^2^)

**Table:**

	*x*	*y*	*z*	*U*_iso_*/*U*_eq_	
Lu	0.896610 (13)	0.396610 (13)	0.103390 (13)	0.00602 (8)	
P	0.95737 (9)	0.37294 (9)	0.33447 (9)	0.0077 (2)	
O1	1.0613 (2)	0.3432 (2)	0.3430 (2)	0.0128 (7)	
O2	1.0325 (2)	0.3642 (3)	0.0522 (2)	0.0188 (8)	
O3	0.9295 (2)	0.3498 (3)	0.2404 (2)	0.0142 (7)	

### Atomic displacement parameters (Å^2^)

**Table:**

	*U*^11^	*U*^22^	*U*^33^	*U*^12^	*U*^13^	*U*^23^
Lu	0.00602 (8)	0.00602 (8)	0.00602 (8)	0.00039 (8)	−0.00039 (8)	−0.00039 (8)
P	0.0097 (5)	0.0051 (6)	0.0082 (6)	0.0017 (5)	−0.0008 (5)	0.0015 (4)
O1	0.0113 (15)	0.0146 (18)	0.0123 (17)	0.0007 (15)	−0.0025 (14)	0.0054 (17)
O2	0.0103 (17)	0.024 (2)	0.022 (2)	0.0016 (18)	0.0015 (16)	−0.0014 (18)
O3	0.0202 (19)	0.0141 (19)	0.0084 (16)	0.0012 (16)	−0.0050 (15)	0.0023 (16)

### Geometric parameters (Å, °)

**Table:**

Lu—O3^i^	2.182 (3)	P—O2^iii^	1.464 (4)
Lu—O3^ii^	2.182 (3)	P—O3	1.481 (4)
Lu—O3	2.182 (3)	P—O1^iv^	1.583 (3)
Lu—O2^i^	2.185 (4)	P—O1	1.594 (3)
Lu—O2^ii^	2.185 (4)	O1—P^v^	1.583 (3)
Lu—O2	2.185 (4)	O2—P^vi^	1.464 (4)
			
O3^i^—Lu—O3^ii^	89.06 (14)	O3—Lu—O2	92.64 (13)
O3^i^—Lu—O3	89.06 (14)	O2^i^—Lu—O2	87.72 (15)
O3^ii^—Lu—O3	89.06 (14)	O2^ii^—Lu—O2	87.72 (15)
O3^i^—Lu—O2^i^	92.64 (13)	O2^iii^—P—O3	118.0 (2)
O3^ii^—Lu—O2^i^	178.26 (14)	O2^iii^—P—O1^iv^	107.3 (2)
O3—Lu—O2^i^	90.59 (14)	O3—P—O1^iv^	111.5 (2)
O3^i^—Lu—O2^ii^	90.59 (14)	O2^iii^—P—O1	109.2 (2)
O3^ii^—Lu—O2^ii^	92.64 (13)	O3—P—O1	106.0 (2)
O3—Lu—O2^ii^	178.26 (14)	O1^iv^—P—O1	103.9 (2)
O2^i^—Lu—O2^ii^	87.72 (15)	P^v^—O1—P	136.9 (2)
O3^i^—Lu—O2	178.26 (14)	P^vi^—O2—Lu	164.9 (2)
O3^ii^—Lu—O2	90.59 (14)	P—O3—Lu	148.2 (2)

Symmetry codes: (i) −*z*+1, *x*−1/2, −*y*+1/2; (ii) *y*+1/2, −*z*+1/2, −*x*+1; (iii) −*z*+1, −*x*+3/2, *y*; (iv) −*y*+5/4, *x*−3/4, −*z*+3/4; (v) *y*+3/4, −*x*+5/4, −*z*+3/4; (vi) −*y*+3/2, *z*, −*x*+1.

## References

[bb1] Bagieu-Beucher, M. (1976). *J. Appl. Cryst.* **9**, 368–369.

[bb2] Bagieu-Beucher, M. & Guitel, J. C. (1978). *Acta Cryst.* B**34**, 1439–1442.

[bb3] Bejaoui, A., Horchani-Naifer, K. & Férid, M. (2008). *Acta Cryst.* E**64**, i48. 10.1107/S1600536808021995 PMC2961903 21202991

[bb4] Boudias, C. & Monceau, D. (1998). *CaRine* CaRine Crystallography, DIVERGENT S.A., Compiègne, France.

[bb5] Bruker (2008). *APEX2*, *SAINT* and *SADABS* Bruker AXS Inc., Madison, Wisconsin, USA.

[bb6] Chudinova, N. N. (1979). *Izv. Akad. Nauk SSSR Neorg. Mater* **15**, 833–837.

[bb7] Durif, A. (1995). In *Crystal Chemistry of Condensed Phosphates* New York and London: Plenum Press.

[bb19] d’Yvoire, F. (1962). *Bull. Soc. Chim* pp. 1237–1243.

[bb8] Farrugia, L. J. (1997). *J. Appl. Cryst.* **30**, 565.

[bb9] Flack, H. D. (1983). *Acta Cryst.* A**39**, 876–881.

[bb10] Hendricks, S. B. & Wyckoff, R. W. G. (1927). *Am. J. Sci* **13**, 491–496.

[bb11] Höppe, H. A. & Sedlmaier, S. J. (2007). *Inorg. Chem. ***46**, 3467–3474. 10.1021/ic0616030 17411030

[bb12] Liebau, F. & Williams, H. P. (1964). *Angew. Chem* **76**, 303–304.

[bb13] Mezentseva, L. P., Domanskii, A. I. & Bondar, I. A. (1977). *Russ. J. Inorg. Chem* **22**, 43–45.

[bb14] Pauling, L. & Sherman, J. S. (1937). *Z. Kristallogr* **96**, 481–487.

[bb15] Rémy, P. & Boullé, A. (1964). *C. R. Acad. Sci* **258**, 927–929.

[bb16] Sheldrick, G. M. (2008). *Acta Cryst.* A**64**, 112–122. 10.1107/S0108767307043930 18156677

[bb17] Smolin, Y. I., Shepelev, Y. F., Domanskii, A. I. & Belov, N. V. (1978). *Kristallografiya*, **23**, 187–188.

[bb18] Yuan, J. L., Zhang, H., Zhao, J. T., Chen, H. H., Yang, X. X. & Zhang, G. B. (2008). *Opt. Mater* **30**, 1369–1374.

